# Characteristics of hypertension and its impact on cognitive functions in older adults: a cross-sectional study

**DOI:** 10.3389/frdem.2024.1486147

**Published:** 2024-11-26

**Authors:** Nivedita Parandiyal, Porimita Chutia, Shashank Saurabh Sinha, Pratyaksha Pandit, Naif Ali Majrashi, Naseem Qureshi, Shailendra Mohan Tripathi

**Affiliations:** ^1^Medical Student, King George's Medical University, Lucknow, UP, India; ^2^Department of Geriatric Mental Health, King George's Medical University, Lucknow, UP, India; ^3^Department of Community Medicine, King George's Medical University, Lucknow, UP, India; ^4^Diagnostic Radiography Technology Department, Faculty of Applied Medical Sciences, Jazan University, Jazan, Saudi Arabia; ^5^School of Medical Science and Research Center, AlFalah University, Faridabad, Haryana, India; ^6^Institute of Medical Sciences, University of Aberdeen, Aberdeen, United Kingdom

**Keywords:** hypertension, cognitive functions, cognition, older adults, late life

## Abstract

**Background:**

Hypertension is a potentially modifiable risk factor for cognitive decline. Understanding the variables of hypertension related to cognitive functions will help in mitigating the risk.

**Objective:**

The study aims to assess the characteristics of hypertension and its effect on cognitive functions in the older adults.

**Methods:**

The study involved 95 hypertensive participants aged 60 years and above from cardiology and medicine outpatient services of a tertiary care hospital from August to October 2022. The characteristics of hypertension and cognitive functions were assessed using semi-structured proforma and Adenbrooke's Cognitive Examination (ACE-III) Hindi version respectively. Further, individual cognitive functions were compared with duration of the hypertension and hypertensive status of the participants. The chi-square test and independent *t*-tests were used and *p* value < 0.05 was considered to be significant.

**Result:**

The mean age of the study population was 68.2 years, the cognitive functions was comparable in terms of age, sex, locality, co-morbidity, and treatment characteristic. Although a significant difference in cognitive functions was present in relation to duration and status of hypertension. Among the individual cognitive domains, a significant difference was observed in attention and fluency domains of cognitive function based on HTN status (*p* > 0.05) but differential effect on cognitive domains was not seen with the duration of HTN. However, there was overall decline in cognitive domains with both hypertension status and the duration of hypertension.

**Conclusion:**

The study highlights association of status of hypertension and its characteristics with cognitive decline.

## Introduction

Worldwide, hypertension is a significant risk factor for cardiovascular disease and mortality (Mills and Stefanescu, 2020). It is also a leading cause of cognitive impairment and dementia (Iadecola et al., 2019). Hypertension affects one-third of adults and two-thirds of the older adults, with higher prevalence in low- and middle-income countries (Mills et al., 2016; Ungvari et al., 2021). In India, the prevalence of HTN is 29.8%. Higher rates are seen in urban population (33.85%) compared to rural population (27.6%) (Anchala et al., 2014). According to the longitudinal aging study of India (LASI), HTN prevalence among people aged 45 and above is 45.9% in India, with only 31.7% having their blood pressure under control (Lee et al., 2022). HTN is characterized by exceeding systolic BP of 140 mmHg and diastolic BP of 90 mmHg (Muntner et al., 2020). Individuals with BP levels below this threshold are considered to have controlled HTN within the hypertensive population (Gillespie and Hurvitz, 2013).

HTN exerts a direct influence on both the vasculature and brain structure, increasing the likelihood of end-organ damage. This raises the risk of cognitive impairment and dementia (Gasecki et al., 2013). HTN has been linked to cognitive impairment through several pathophysiological mechanisms, including reduced cerebral blood flow, rarefaction of microvasculature, disruption of the blood-brain barrier (BBB), oxidative stress, and impaired neurovascular coupling. Moreover, it is worth noting that cerebral micro-hemorrhages, lacunar infarcts, and white matter (WM) injuries are frequently observed in individuals with both hypertension and dementia (Ungvari et al., 2021). Mid-life hypertension carries a lifetime risk of 20%−54% of developing dementia (McGrath et al., 2017). Previous studies have shown that the risk of vascular events and strokes doubles with uncontrolled HTN and subsequently increases the risk of dementia. Chronic uncontrolled hypertension reduces elasticity and increases vascular resistance, resulting in higher pulse pressure and increased risk for cognitive impairment due to WM lesions and transient hemorrhage (Beason-Held et al., 2007; Mitchell, 2008). HTN is a recognized risk factor for both vascular cognitive impairment and Alzheimer's Disease (AD), which often co-exist and constitute the majority of cases of dementia (Wei et al., 2018; Iadecola and Gottesman, 2019). Incidental neuroimaging and post-mortem studies on people with AD reveal vascular pathology in approximately one-third of the patients (Snowdon et al., 1997; Schneider et al., 2004).

Considering the modifiability of HTN in the early stages and understanding its characteristics, we can elucidate its role in cognition. This will further aid in understanding the mechanisms involved in developing cognitive impairment and exploring newer therapeutic targets. Although previous studies highlighted mid-life hypertension as a common risk factor for developing dementia, late-life hypertension is less studied, and the results are variable. Moreover, several studies in the past have tried to explore the impact of controlled HTN on halting the progression of cognitive decline but the evidence is inconsistent (Mishra et al., 2020). The current study aimed to explore the characteristics of HTN and its impact on cognitive functions in the older adults. It could contribute to existing knowledge and enhance clinical practice. This knowledge could guide healthcare professionals in implementing preventive measures, optimizing hypertension management, and developing interventions to preserve cognitive health in older adults with hypertension. Ultimately, this research could improve the quality of life and overall wellbeing of older adults by addressing the complex relationship between hypertension and cognitive functions.

## Materials and methods

### Participants

The study involved the enrolment of 95 participants with a diagnosis of hypertension and their demographics and clinical data were obtained from the Cardiology and Medicine Outpatient Department (OPD) at King George's Medical University (KGMU) in Lucknow, spanning the period of September through October 2022. The selection of participants was done using a purposive sampling technique. This cross-sectional study was conducted after obtaining approval from the Institutional Ethical Committee, with reference number XIPGTSCIB/P3. The study was conducted in accordance with the ethical standards set forth by the institutional and national committees responsible for human experimentation, as well as the Helsinki Declaration of 1975, which was subsequently revised in 2013.

### Inclusion and exclusion criteria

The inclusion criteria for both participants were: (1) hypertensive patients of age 60 years or above; (2) participants with at least 5 years of formal education who can read and write. The exclusion criteria for both participants were: (1) patients with severe vision and hearing impairment; (2) patients with any known psychiatric and cognitive disorders; and (3) Severely ill and uncooperative participants.

### Sample size

According to a previous study (Mekala et al., 2020), the occurrence of cognitive impairment within the North Indian population is reported to be 8.8% (Sengupta et al., 2014). The calculation of the sample size (n) is determined using the formula; *n* = (z_α/2_)^2^^*^P(1-p)/MOE^2^. Given a confidence level of 95%, a proportion (p) of 0.088, a margin of error (MOE) of 0.06, and a value of zα/2 equals 1.96, the sample size (n) was determined to be 84. After accounting for a non-responsive rate of 10%, the final sample size is determined to be 92, which was rounded up to 95.

### Clinical measures (hypertension and cognition)

The participants were evaluated using a semi-structured proforma for socio-demographic and clinical details. Patients were analyzed based on their current BP, HTN duration, and status (Controlled and uncontrolled HTN). The definition of controlled hypertension in hypertensive individuals is SBP <140 mm Hg and DBP <90 mm Hg (Gillespie and Hurvitz, 2013). They were asked the following questions about hypertension: “When were they first diagnosed with hypertension?” for determining the age of onset of HTN, “Are they under treatment for HTN, and if yes, how long have they been under medication?” and “Are they taking medications regularly?” The family history of patients and the history of any other medical co-morbidity were also taken. The treatment in this study refers to subjects on antihypertensive medications at any point in time after the diagnosis of HTN. The cognition of participants was evaluated using the Adenbrooke's Cognitive Examination (ACE) III Hindi version (Mekala et al., 2020). The ACE scale assesses the cognition of a person using five domains: attention, fluency, memory, language, and visuospatial. Each domain carries a different score, and a higher total score implies better cognition. Using the total ACE score, a cut-off of 71 was considered for classifying the subjects as cognitively normal, and impaired (Bajpai et al., 2020).

### Data analysis

Data was collected using a semi-structured proforma and the ACE III Hindi version. This data was tabulated in Microsoft EXCEL, and statistical analysis was done on the Statistical Package for Social Sciences (SPSS), version 24, IBM, Chicago, USA. Descriptive data were represented as frequency percentage, mean, standard deviation (SD), or proportion. Probability (p) was calculated to test for statistical significance at the 5% level of significance. The association between categorical variables was tested using the Chi-Square test. However, differences between continuous variables were tested using the student *t-*test. A minimum 95% confidence interval or *p*-value <0.05 was considered statistically significant.

## Results

### Participant characteristics

The current cross-sectional study included a sample of 95 participants diagnosed with HTN with a mean age of 68.2 ± 3.29 years. The mean age of onset of HTN was 62.64 ± 3.69 years and the mean duration of HTN was 5.53 ± 1.86 years in the participants. No statistically significant differences in cognitive functions were observed based on gender, age, and residence areas (urban or rural) (all *p* > 0.80). A total of 90 subjects were on treatment; among 77 subjects with uncontrolled HTN, 4 were not on treatment. Statistically significant differences in cognitive functions were observed with the duration of HTN, Blood pressure measures (Systolic and Diastolic blood pressure), HTN status, and duration of treatment, as indicated by chi-square tests (*P* < 0.05). Table 1 displays the demographic and clinical characteristics associated with cognitive functions.

**Table 1:** Comparison of socio-demographic and clinical characteristics of subjects with Hypertension with and without cognitive impairment.

**Variable**		**Cognitive function**			***p*-value**
		**Impaired** ***n*** = **24**	**Unimpaired** ***n*** = **71**	**Total** ***n*** = **95**	
**Socio-demographic variables**					
Gender	Male	20 (25.39%)	59 (74.68%)	79	0.979
	Female	4 (25%)	12 (75%)	16	
Mean age in years ± SD		68.1 ± 2.12	68.4 ± 3.23	68.2 ± 3.29	0.700
Residence	Urban	14 (26.41%)	39 (73.58%)	53	0.772
	Rural	10 (23.80%)	32 (76.19%)	42	
**Clinical variables**					
Hypertension status	Controlled	1 (5.55%)	17 (94.44%)	18	**0.033**
	Uncontrolled	23 (29.81%)	54 (70.12%)	77	
Hypertension duration	<5 years	14 (38.88%)	22 (61.11%)	36	**0.017**
	>5 years	10 (16.94%)	49 (83.05%)	59	
Systolic blood pressure		159.8 ± 23.7	156.4 ± 23.2	157.31 ± 23.29	0.900
Diastolic blood pressure		83.67 ± 17.5	83.07 ± 11.69	83.22 ± 13.31	**0.001**
Family history of HTN	Yes	5 (29.41%)	12 (70.58%)	17	0.664
	No	19 (24.35%)	59 (75.64%)	78	
Comorbidities	Yes	17 (24.63)	52 (75.36)	69	0.819
	No	7 (26.92)	19 (73.07)	26	
Duration of treatment (*n* = 90)	<1 year	6 (40%)	9 (60%)	15	**0.009**
	1–3 years	4 (25%)	12 (75%)	16	
	3–5 years	4 (80%)	1 (20%)	5	
	>5 years	9 (16.66%)	45 (83.88%)	54	
Regular medication (*n* = 90)	Yes	17 (23.61)	55 (76.38%)	72	0.398
	No	6 (33.33%)	12 (66.66%)	18	

Statistics: chi-square test and independent *t*-test, *p*-value <0.05 is considered as statistically significant denoted by bold values, SD, standard deviation.

### Comparison of cognitive functions based on the status of HTN

There was a significant difference in overall cognitive function and in domains of attention and fluency according to HTN status with *p*-values of 0.04, 0.01, and 0.02 respectively (Table 2).

**Table 2:** Comparison of cognitive subdomains of ACE score based on the status and duration of Hypertension among the study population.

**Cognitive domains**	**Hypertension status** ***N = *** **95 (Mean** ±**SD)**		***p*-value**	**Hypertension duration** ***N = *** **95 (Mean** ±**SD)**		***p*-value**
	**Controlled**	**Uncontrolled**		<**5 years**	>**5 years**	
Attention	17.2 ± 1.3	16 ± 2.5	**0.01**	15.8± 2.6	16.5 ± 2.2	0.142
Memory	16.8 ± 4	15 ± 5.1	0.19	14.3 ± 5.4	16.2 ± 4.6	0.067
Fluency	8.8 ± 2.2	7.5 ± 2.1	**0.02**	7.2 ± 2.1	8.1 ± 2.2	0.058
Language	24.9 ± 1.2	24.2 ± 2.3	0.08	23.8 ± 2.8	24.7 ± 1.7	0.053
Visuospatial	13.7 ± 1.8	13.4 ± 2.3	0.53	13.3 ± 2.4	13.5 ± 2.2	0.801
Overall score	81.5 ± 6.6	76.2 ± 10.7	**0.04**	74.5 ± 11.7	78.9 ± 9.1	**0.044**

Statistics: independent *t-*test, *p*-value <0.05 is considered as statistically significant denoted by bold values, SD, standard deviation.

### Comparison of cognitive functions based on the duration of HTN

There was a significant difference in overall cognitive function score based on the duration of HTN (<5 years and >5 years) with p 0.04 (Table 2). However, individual cognitive domains did not differ based on the duration of HTN.

## Discussion

HTN and cognitive impairment are the two most frequently occurring conditions in the older adults (Iadecola et al., 2019) and the current study is the first to investigate the characteristics of HTN and its impact on cognitive functions in older adults of India. Notably, 95.8% of people with cognitive impairment in this study had uncontrolled HTN. People with uncontrolled HTN had poorer cognitive functions compared to people with controlled hypertension. This is in accordance with a recent study in which hypertensive individuals with uncontrolled BP had more cognitive impairment in comparison to controlled hypertensives (de Menezes et al., 2021). Our findings also support another study reporting chronic uncontrolled HTN can lead to cognitive impairment as it increases the risk of white matter lesions by increasing vascular resistance and vessel wall stiffening and it can be inferred that the risk of cognitive impairment was higher in uncontrolled hypertensives than in controlled hypertensives (Mitchell, 2008). Moreover, people with <5 years of duration of HTN had more chances of developing cognitive impairment in comparison to hypertensives of >5 years duration (38.88% vs. 16.94%) which is not in agreement with the studies reporting midlife hypertensives are at increased risk cognitive impairment (Launer et al., 1995; Livingston et al., 2020). Moreover, a recent study reported minimal effect of late-life hypertension on cognition (Moll and Woodard, 2022). However, a few other studies have reported an association of late-life hypertension with cognitive impairment in older adults and suggested that the association between cognition and hypertension is age-dependent (Wei et al., 2018).

HTN is a modifiable risk factor for cognitive impairment and it can be presumed that it can be modified by antihypertensive treatment at early stages to reduce the risk of cognitive impairment (Wei et al., 2018). Notably, the people with regular medication for a duration of 3–5 years had more chances of developing cognitive impairment in comparison to people who were commenced on antihypertensive medications recently (<3 years) or more than 5 years ago which contradicts the findings of a meta-analysis suggesting reduced risk of incident dementia with antihypertensive use as antihypertensive use for 3–5 years had higher impairment in cognition (Ding et al., 2020). It suggests that antihypertensive use can modify the disease or reduce the risk of dementia for the initial few years and causes cognitive decline later as low and high blood pressure both can increase the risk of cognitive impairment (Ding et al., 2020) and blood pressure variability in older people increases the risk of dementia (Razay et al., 2009; Oishi et al., 2017). However, people on antihypertensive treatment with more than 5 years duration of hypertension had less chance of cognitive impairment and this supports the findings that early commencement of treatment with antihypertensive medication may be associated with better cognitive functions later in life (Mogi, 2022).

On further evaluation of individual cognitive domains with HTN status, the domains were differentially affected as the attention and fluency differ significantly in controlled and uncontrolled hypertensives. The uncontrolled hypertensives had lower scores on attention and fluency in comparison to controlled hypertensives and it seems like attention is primarily affected cognitive domain with uncontrolled hypertension as fluency is a cognitive skill directly associated with attention and working memory (Francisco et al., 2019). A deficit in attention due to uncontrolled hypertension may lead to a deficit in verbal fluency and this contradicts the earlier findings that hypertension affects cognitive processing speed and episodic and working memory but not attention (Saxby et al., 2003). However, the differential effect on specific cognitive domains was not seen with the duration of hypertension, and an overall deficit in cognitive scores was found in the people with a lesser duration of the illness in this study which is not per a previous study reporting deficit in only verbal memory with duration of hypertension (Zhou et al., 2022). Our study also contradicts the findings that midlife hypertension has a greater risk for cognitive impairment in comparison to late-life hypertension (Livingston et al., 2020).

## Limitations of the study

The current study has several limitations including this study was conducted with a limited sample size, suggesting that it may be prone to overestimating the magnitude of the effect size and the sample population may not be representative of the general population as this is a hospital-based study. Further, a multi-centric study with a large sample size is required to overcome the limitations of the study. Moreover, longitudinal studies are needed to find the association between the trajectory of cognitive decline and characteristics of hypertension in subjects with hypertension.

## Conclusion

This study highlighted the impact of hypertension characteristics including hypertension status (controlled and uncontrolled) and duration of hypertension with cognitive functions. Uncontrolled hypertensives had poorer cognitive functions in comparison to controlled hypertensives. Moreover, there was a differential impact of uncontrolled hypertension on cognitive domains as attention and fluency were more affected in comparison to other cognitive domains. Interestingly, those with a shorter duration of HTN had lower cognitive scores than those with a longer HTN duration.

## Data Availability

The raw data supporting the conclusions of this article will be made available by the authors, without undue reservation.
